# Brassinosteroids and the Tolerance of Cereals to Low and High Temperature Stress: Photosynthesis and the Physicochemical Properties of Cell Membranes

**DOI:** 10.3390/ijms23010342

**Published:** 2021-12-29

**Authors:** Iwona Sadura, Anna Janeczko

**Affiliations:** Polish Academy of Sciences, The Franciszek Górski Institute of Plant Physiology, Niezapominajek 21, 30-239 Kraków, Poland

**Keywords:** acclimation, brassinosteroids, cereals, hardening, high temperature, frost

## Abstract

Cereals, which belong to the *Poaceae* family, are the most economically important group of plants. Among abiotic stresses, temperature stresses are a serious and at the same time unpredictable problem for plant production. Both frost (in the case of winter cereals) and high temperatures in summer (especially combined with a water deficit in the soil) can result in significant yield losses. Plants have developed various adaptive mechanisms that have enabled them to survive periods of extreme temperatures. The processes of acclimation to low and high temperatures are controlled, among others, by phytohormones. The current review is devoted to the role of brassinosteroids (BR) in cereal acclimation to temperature stress with special attention being paid to the impact of BR on photosynthesis and the membrane properties. In cereals, the exogenous application of BR increases frost tolerance (winter rye, winter wheat), tolerance to cold (maize) and tolerance to a high temperature (rice). Disturbances in BR biosynthesis and signaling are accompanied by a decrease in frost tolerance but unexpectedly an improvement of tolerance to high temperature (barley). BR exogenous treatment increases the efficiency of the photosynthetic light reactions under various temperature conditions (winter rye, barley, rice), but interestingly, BR mutants with disturbances in BR biosynthesis are also characterized by an increased efficiency of PSII (barley). BR regulate the sugar metabolism including an increase in the sugar content, which is of key importance for acclimation, especially to low temperatures (winter rye, barley, maize). BR either participate in the temperature-dependent regulation of fatty acid biosynthesis or control the processes that are responsible for the transport or incorporation of the fatty acids into the membranes, which influences membrane fluidity (and subsequently the tolerance to high/low temperatures) (barley). BR may be one of the players, along with gibberellins or ABA, in acquiring tolerance to temperature stress in cereals (particularly important for the acclimation of cereals to low temperature).

## 1. Introduction: Plant Steroid Hormones—Brassinosteroids

**Figure 1:**
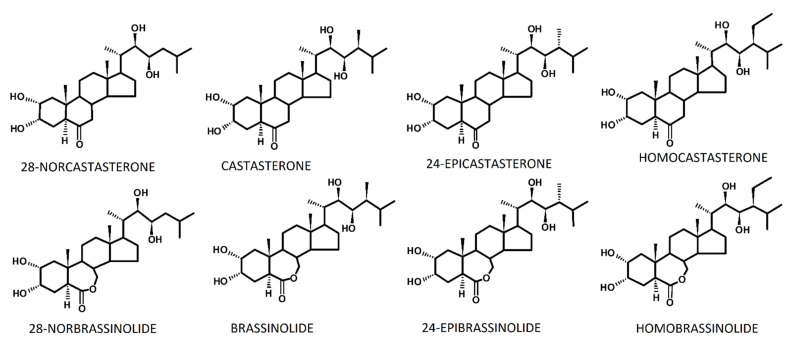
Structural formulas of the selected BR: C_27_ (28-norcastasterone, 28-norbrassinolide), C_28_ (castasterone, brassinolide and 24-epicastasterone, 24-epibrassinolide) and C_29_ (homocastasterone, homobrassinolide). Figure was prepared in ChemSketch program and is based on the chemical structures of BR that are available in an article of Bajguz and Tretyn [3].

Brassinosteroids (BR) are plant steroid hormones that are structurally similar to the steroid hormones that are found in animals (corticosteroids, estrogens, androgens, progesterone) and insects (ecdysteroids). BR were first isolated from rape pollen (*Brassica napus* L.) in the 1970s. The first BR, which was called brassinolide, was then identified [1]. Currently, this group consists of 81 compounds that differ in their structure [2], which in higher plants and algae occurs in both a free and bound form (conjugates with fatty acids or sugars). BR are present in plants in amounts of nano- and picograms per gram of fresh weight. The discussed chemical compounds are characterized by a polycyclic carbon skeleton of 5α-cholestane and the differences in their structures result from the presence of various functional groups and their location within the cyclic A and B rings and the carbon chain [3,4]. Due to the number of carbon atoms in a molecule, there are three main BR groups: C_27_, C_28_, and C_29_. The precursors in the biosynthesis of all BR are sterols. In the case of BR C_27_, the precursor is cholesterol; for BR C_28_, it is campesterol and 24-β-methylcholesterol and for BR C_29_, it is sitosterol [5]. The structural formulas of the selected BRs are presented in Figure 1.

The first biosynthetic pathway was described for BR C_28_ [6,7,8]. The intermediate metabolites in this pathway are cathasterone, teasterone, typhasterol, and castasterone in the early C6 oxidation pathway and 6-deoxocathasterone, 6-deoxoteasterone, 6-deoxotyphasterol and 6-deoxocastasterone in the late C6 oxidation pathway [7]. In the case of BR with different structures, the pathways of their biosynthesis can be modified and their simplified versions are presented in the publication of Sadura and Janeczko [5]. There are known mutants with disturbances in BR biosynthesis, mainly in the C_28_ biosynthetic pathway. These mutants are characterized by a deficiency of BR, in particular, castasterone and brassinolide [4,9,10,11,12]. The current work mainly discusses the results that were obtained for barley with disturbances in BR biosynthesis (BW084 and 522DK) and BR perception (BW312). 

BW084 and BW312 are Near-Isogenic Lines of the Bowman cultivar. BW084 (*brh13.p*) carries the C2562T missense mutation in the *HvCPD* gene that encodes barley C-23α-hydroxylase cytochrome P45090A1 (CYP90A1), which catalyzes the early stages of BR biosynthesis. Point mutation C2562T causes the substitution of the highly conserved amino acid residue (Pro-445 to Leu). Pro-445 is located within the highly conserved heme binding site in the C-terminal part of the HvCPD [10]. The mutant is characterized by a lower content of BR (brassinolide and castasterone can even be below the detection limits) [13]. The BW312 (*ert-ii.79*) NIL is defective in BR perception. The sequencing of *HvBRI1* revealed that it is characterized by a double substitution (C1760A and C1761A). The substituted amino acid residue (Thr-573 to Lys) is located in the steroid-binding island domain of the receptor BRI1. The charged Lys-573, which is introduced to the hydrophobic active site surrounding the residues, destroys the charge neutrality and is suspected of preventing BR binding [10]. As an effect of the mutation, BW312 is characterized by an increased content of BR [13]. 

The 522DK mutant was obtained via the chemical mutagenesis of the cultivar Delisa. The mutant is characterized by a G > A substitution at position 1130 of the *HvDWARF* transcript [9], and at position 3031 in the gene sequence [14], which changes the valine-341 residue into isoleucine. The substituted valine-341 is a highly conserved residue that is present in a similar position in the homologous DWARF polypeptides from barley, rice, *Arabidopsis*, and tomato. The *HvDWARF* gene encodes the brassinosteroid C6-oxidase, which is involved in BR biosynthesis (catalyzing biosynthesis of castasterone). That is the reason for the lower content of castasterone [14] and other BR [13].

The enzymes that are responsible for BR biosynthesis belong to the cytochrome P450 family and are located in the endoplasmic reticulum membrane [15,16]. To date, it is known that the BR signal transduction pathway starts from the transmembrane receptor BRI1 (Brassinosteroid insensitive-1) and it has been described quite well [17,18,19,20,21]. Although it was found that exogenous treatment with BR stimulates stem elongation and cell division in plants in the 1970s [1], they were considered to be a new class of phytohormones in the 1990s. The genes that are responsible, among others, for the biosynthesis and perception of BR were discovered and it was found that the mutation that is associated with the loss of the function of these genes leads to significant disturbances in plant growth (including a short hypocotyl, dwarfism in seedlings and mature plants and dark green shortened leaves) [22]. However, it is already known that the action of BR is multidirectional and that it also concerns the regulation of plant development and their response to stress factors [23,24,25]. The multidirectional activity of BR includes changes in the membrane physicochemical properties (changes in the proportion of unsaturated fatty acids and the possibility of BR incorporation into the membranes, which modifies its properties), regulates the expression of some proteins and genes (*HSP, COR*) and regulates the plant metabolism through other hormones or signaling molecules (ABA, JA, or hydrogen peroxide) [5,24,25,26,27,28]. BR are also important factors in regulating photosynthesis, for example, they can influence PSII efficiency [5,23,26,28].

The occurrence and physiological activity of BR in cereals are discussed in more detail in a chapter in Janeczko [29]. On the other hand, the review of Sadura and Janeczko [5] discusses the role of BR in the response of plants to temperature stress. However, the review of the literature on BR and temperature stress that was conducted in the study of Sadura and Janeczko [5] indicated the existence of a relatively small number of studies in which mutants with BR biosynthesis or perception disturbances were used for the research [30,31,32,33]. Additionally, none of these four works were about research on cereals. Thus, many conclusions about the role of BR in the plant response to low/high-temperature stress in the case of cereals, have been made according to a research model that uses exogenous BR. In some cases, this state of knowledge may require some verification and/or confirmation, for example, in research using mutants. This review focuses mainly on the achievements in the study of the acclimation processes to frost and high temperature in barley (*Hordeum vulgare* L.) and its mutants with an impaired BR biosynthesis (a reduced content of BR from the C_28_ and C_29_ groups) and an impaired perception of BR (a defect of the BRI1 receptor and an increased content of BR from the C_28_ group) [13,34,35,36,37] in recent years. The knowledge that was gained in these studies is discussed against the background of the results of other/previous works on cereals, especially those that included experiments where BR were applied exogenously. Although there are available research articles and reviews devoted to the acclimation of the photosynthetic apparatus in heat/cold/frost-exposed cereals [38,39,40,41,42,43,44,45], much less attention has been paid to the hormonal regulation (particularly regarding BR) of the adjustment of photosynthetic apparatus to temperature stress. Similarly, the importance of the cell membranes in the acclimation of cereals and other species is unquestionable [46,47,48], while the impact of hormones on the membrane properties in these aspects has been less described [49]. That is why our review is focused on the process of photosynthesis and the physicochemical properties of membranes, which are extremely important for the acclimation of plants to temperature stress and the possible regulatory role of the plant hormones, brassinosteroids.

## 2. Temperature Stress and Cereal Production—Significance of the Plant Hardening/Acclimation Process

Plants are exposed to various environmental factors both biotic and abiotic [50]. Among the abiotic stresses, temperature stresses are a particularly serious problem for plant production [50,51]. Cereal species such as corn are extremely sensitive to cold. In turn, frost, especially with insufficient snow cover in the fields (and additionally preceded by a period of higher temperatures with a dehardening effect), can cause significant yield losses of winter cereals. According to Statistics Poland [52], in 2020, approx. 2.3 thousand ha of winter cereals were qualified for plowing due to frost injuries in Poland. However, in 2016, many more winter cereals were qualified for plowing, i.e., approx. 400 thousand ha of the winter cereals that had been sown. In 2020, in Europe, similar to Poland, the injuries of winter cereals were estimated as being minor [53] due to a mild winter, while in 2012, the injuries were estimated at a higher level [54]. 

In turn, the prolonged period of high temperatures and strong sunlight on crops in recent years also had a decisive impact on the yield of cereal plants. Particularly, drought conditions combined with high temperature is dangerous. The frequency and intensity of droughts in Poland have become an increasingly important problem in recent years [55]. Over the last hundred years, the average annual air temperature in Poland and other countries in the world has increased [56,57]. In recent years, the monthly values of the climatic water balance (CWB) in spring and early summer have exhibited a clear downward trend, which coincides with the period of the maximum demand for water by crops including cereals. These trends were manifested by the extreme drought in 2006 as a result of which, the average national yields of some crops fell by as much as 30% and the financial losses were estimated at several billion PLN [58]. This problem also concerns other European countries [59,60,61,62]. Between 1976 and 2006, the number of areas and people that were affected by droughts increased by 20% and the total costs of droughts were estimated at EUR 100 billion [63]. In the case of cereals, the occurrence of drought during the critical period—in the heading stage of the plants [64,65]—leads to particularly high yield losses. As was mentioned above, this effect can be significantly intensified in the event of extremely high temperatures during the same period. 

Plants have developed various adaptive mechanisms that have enabled them to survive periods of extreme temperatures. Frost tolerance in winter cereals (wheat, rye, barley) increases as a result of the metabolic changes that occur during the cold hardening (acclimation) period. Among others, in the European regions, cold periods during autumn (especially temperatures of 2–5 °C) harden winter crops, which increases their tolerance to frost during winter. Frost tolerance increases with the duration of the acclimation time [66] and a cold period that lasts 3–6 weeks is considered to be sufficient. Spring cultivars also have the ability for cold acclimation, which is useful in the event of temperature anomalies (frost incidents) in the spring. On the other hand, some cereal species like maize, which originate from warm regions of the world, have a poor ability to acclimate to low temperatures and are even sensitive to a mild chill [67]. 

The metabolic changes that occur during the cold-hardening process are quite well known and include the inhibition of plant growth, a decrease in tissue hydration, a change in the lipid composition and membrane structure (an increase in the content of unsaturated fatty acids in the membrane lipids), changes in the protein content, among others, an increase in the accumulation of late embryogenesis abundant proteins (LEA), anti-freezing proteins (AFP), and cold-shock proteins (CSP) [68]. Other changes include an increase in the level/activity of enzymes, e.g., those that are associated with the biosynthesis of the osmoprotectants and antioxidants [69] and an increase in the concentration of soluble sugars [70,71,72]. During hardening, a low temperature causes the activation of the genes that perform various functions, including the genes that are responsible for the signal transduction that is caused by a low temperature. Among these genes are the *C-repeat Binding Factors* (*CBFs*), *GA2ox1, GA2ox3*, and *GA2ox6, repressor of GA3* (*RGL3*) or *Cold-Responsive Genes (COR)* [73,74,75]. The coordinated interplay between these genes and the environment enables the plant metabolism and morphology to be prepared for changing temperature conditions. The transcription factor gene, which is crucial for hardening, i.e., C-repeat binding factor (CBF) is the most important. Cold-induced or the constitutive expression of *CBF1* reduces the level of bioactive gibberellins (accompanied by an increase in *GA2ox3* and *GA2ox6* transcripts), which results in a change in the stability of the DELLA proteins and their accumulation. Moreover, chill and CBF increase the transcription levels of the *Repressor of GA3* (*RGL3*). Low temperature also increases the *GA2ox1* expression levels through a mechanism that is independent of *CBF1*. As a result of the accumulation of DELLA, plant growth slows down, while an increase in the level of active gibberellins in spring is responsible for promoting their growth [76,77]. CBF also induces the expression of the cold-responsive genes (COR), which are important in the processes of plant acclimation to low temperatures [73,74]. The fundamental role of the COR is to protect plant cells against water loss [73]. Both the CBF and COR transcription factors help plants to achieve frost tolerance, among others, by accelerating the osmolyte synthesis or remodeling the cell membranes [73,75]. 

In turn, plants that are grown at higher temperatures (including acclimation, e.g., at 27 °C) may have an increased tolerance to the effects of a more extreme high temperature, e.g., 40–45 °C. However, unlike hardening to low temperatures, an increased tolerance to high temperature is acquired by plants (depending on the species) after a shorter period of time, even as little as a few hours [78] to 7–14 days [38,79] of exposure to a higher temperature. The mechanism of plant acclimation to a high temperature includes changes in the composition and integrity of the membranes, including an increase in the saturated fatty acid content that limits excessive increases in membrane fluidity [75,80,81]. Other changes include the activation of the signaling pathways that involve the signaling molecules (e.g., calcium ions, nitric oxide, hydrogen peroxide) or the signaling pathways that are dependent on the phytohormones. Signaling molecules/phytohormones contribute to the activation of the heat shock transcription factors (*HSF*) that induce changes in the expression of the heat-responsive genes (*HR*) [75].

As was mentioned earlier, the processes that plants have developed to acclimate to low and high temperatures are controlled by various metabolic regulators, including phytohormones such as cytokinins [32,82,83], gibberellins [32,76,83,84,85], and auxins [32,83,86]. Recently, much more is being revealed about the role of BR.

## 3. The Impact of Brassinosteroids on the Tolerance of Cereals to Low and High Temperatures

**Figure 2:**
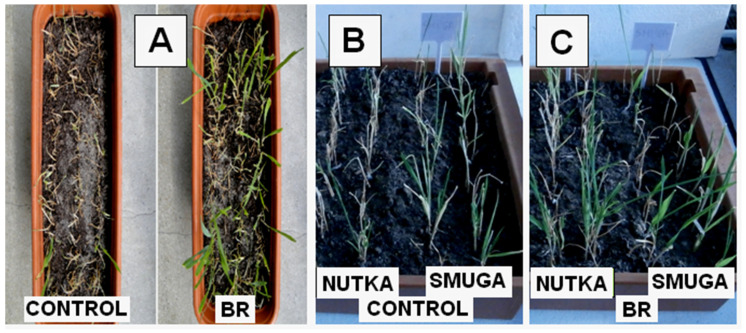
After-frost regrowth of the winter cereals that had been pretreated with brassinosteroids (BR). (**A**) Winter rye plants of the cold-acclimated control after exposure to −17 °C (left) and plants that had been sprayed with BR (24-epibrassinolide) before cold acclimation and then exposed to −17 °C (right) (based on Pociecha et al. [88], photo courtesy of E. Pociecha). Winter wheat (cultivars Nutka and Smuga): control plants that had been cold acclimated and then exposed to −12 °C (**B**), plants that had been sprayed with BR (24-epibrassinolide) before cold acclimation and then exposed to −12 °C (**C**) (based on Janeczko et al. [89]).

One of the first studies concerning the role of BR in improving the cold and frost tolerance in cereals was performed using an exogenous application of these hormones. Singh et al. [87] reported that in cold-stressed seedlings of maize (*Zea mays* L.) (growing in a net house—max. temp. 17.6–24.5 °C, min. temp. 2.8–7.4 °C, 21 days), there was a decrease in both plant height (35%) and fresh mass (24%). The application of 1μM of 24-epibrassinolide increased plant height and fresh as well as dry biomass by about 15, 36, and 2%, respectively, compared to the plants that had not been subjected to steroid treatment. As was mentioned earlier, in winter cereals, cold has a hardening effect that improves their tolerance to frost. In this case, the application of BR before cold hardening (acclimation) additionally increased the frost tolerance. Pociecha et al. [88] studied winter rye plants (*Secale cereale* L.; cv. Dańkowskie Złote—winter-resistant and Stach—moderately winter-resistant) that had been acclimated at 4 °C for three and six weeks. The plants that had been sprayed with 24-epibrassinolide (0.25 mg∙dm^−3^) before the six-week-cold acclimation had fewer frost injuries and a better survival rate (better plant regrowth) than the plants that had not been pretreated with the steroid (Figure 2A). The application of BR also improved the frost tolerance in winter wheat (*Triticum aestivum* L.) [89]. Wheat seedlings that had been sprayed with 24-epibrassinolide (0.25 or 0.05 mg dm^−3^), cold-acclimated, and then exposed to −12 °C (24 h) also had a better survival rate than those that had not been treated with the steroid (Figure 2B,C). Interestingly, another exogenously applied BR (castasterone) was not effective [89], which suggests a role of the chemical structure of BR in their protective activity against frost. 

Based on the presented results, it can be presumed that if the increase in the amount of BR (as a result of their exogenous application) increases the tolerance to low temperatures, then in mutants with disturbances in the biosynthesis and perception of BR, the frost tolerance should be weaker. In order to verify this assumption, studies were conducted on barley plants of the Bowman cultivar (wild type) and its near-isogenic lines (NILs): BW084 (with disturbances in the early stage of the BR biosynthesis pathway), BW312 (with a disturbance in BR signaling) [10] as well as the Delisa cultivar (wild type) and its mutant 522DK, which had been obtained via chemical mutagenesis and had proven disturbances in the late stage of the BR biosynthesis pathway [9]. These studies proved the occurrence of frost tolerance disorders in the cold-acclimated wild-type barley plants as well as in the NILs or mutant [13]. After acclimation at 5 °C, the plants of the wild-type Bowman and its NILs BW084 and BW312 and the wild-type Delisa and its mutant 522DK were characterized by a similar, high survival rate at −6 °C (8.5–9.0 points on the 9-point Larsen scale [13]). At −8 °C, the Bowman cultivar maintained a rather high survival rate (7.5 points), while the Delisa cultivar was much less tolerant to frost (3 points). In the case of the wild type with a higher frost tolerance (Bowman), a BR deficit or disturbances in the perception of BR that occurred in the BW084 and BW312 lines were indeed associated with a reduced survival rate in frost (up to approx. 4.5–6.0 points [13]). However, in the case of the frost-sensitive wild-type Delisa, the BR deficiency had no effect on any further decrease in the plant tolerance to frost [13].

As for the role of BR in the tolerance of cereals to a high temperature, one of the first studies was conducted for the barley (cultivar Sezam) [90]. The leaves of seedlings were injected with BR (24-epibrassinolide) and then exposed to heat shock (42 °C, 3 h). A transient protective effect of BR was observed for photosynthesis (PSII efficiency) in the seedlings. However, there was no after effect of BR (at a concentration of 0.05 mg dm^−3^) when combined with heat shock on plant yield compared to the control that had not been treated with BR.

In another study on rice (*Oryza sativa* L.), a temperature of 40/30 °C (seven days) reduced the shoot and root fresh weight and leaf area compared to the control that had been grown at the optimal temperature. This effect was limited by 24-epibrassinolide (10^−8^ M) [91]. In field research that was conducted on rice during the hot season (February to May 2012, Thailand), 24-epibrassinolide increased the number of filled seeds per panicle by 16% and increased the seed mass by 35% [79]. Additionally, for the same species, Fahad et al. [92] reported that a temperature of 35/32 °C (d/n) (12 h/12 h) caused a reduction in pollen fertility, anther dehiscence, germinated pollen on the stigma, and total pollen count on the stigma compared to the non-stressed plants. The plants that had been treated with a mixture of the regulators that also contained BR had significantly higher values of the aforementioned parameters compared to the plants that were stressed, but that had not been sprayed.

It could be supposed that if the exogenous application of BR increases the tolerance to a high temperature, plants with a deficiency in BR or disturbances in BR signaling should be more susceptible to that stress. Interestingly, the research on BR-defective barley plants that was conducted by Sadura et al. [13] showed that after acclimation at 27 °C (seven days), all of the barley plants with a BR deficiency or disturbances in the perception of BR were significantly more tolerant to high-temperature stress (38 °C and 45 °C) than their wild-type Bowman and Delisa cultivars [13]. After exposure to a high temperature, these plants had significantly fewer injuries of their leaves as well as fewer cell membrane injuries. Thus, the observed effect would be opposite to that of the experiments when the BR were applied exogenously. However, it is worth noting that the model of the experiment that was used by Sadura et al. [13] was different as a weekly acclimation period at 27 °C was used. On the other hand, it is known that semi-dwarf plants might have a greater tolerance to a high temperature because it is usually accompanied by a drought effect that is associated with increased transpiration [93]. A smaller leaf area can limit this effect. 

In conclusion, in the case of the studies on the importance of BR for the frost tolerance of plants, both experiments with exogenously applied BR and studies on plants with disturbances in the BR biosynthesis/perception agree that BR might be an important player in the acquisition of frost tolerance in cereals. However, in the case of the role of BR for the tolerance of cereals to a high temperature, the results of the tests on BR-mutants are not consistent with the results that have been obtained in studies with exogenously applied BR. This might suggest a different model of BR action on the plant metabolism after the exogenous application than in the case of BR-mutants under high-temperature stress (where other phenomena such as a mutant’s architecture might be dominant). In this context, the results of the research on the BR barley mutants that were described in the work Pociecha et al. [94] are worth discussing. As was previously mentioned, the occurrence of warm breaks during autumn, which results from climate change has been an increasing problem in recent years. Normally in autumn, winter cereals acquire frost tolerance via their exposure to cold (cold acclimation). Warm breaks cause the reverse effect (deacclimation/dehardening), which lowers the frost tolerance in cereals [95]. Pociecha et al. [94] observed an interesting phenomenon that plants with decreased BR levels or a BR receptor defect had a very high ‘tolerance to dehardening (deacclimation)’ and maintained high frost tolerance after dehardening periods of warm weather. After deacclimation, the BW084 plants (BR biosynthesis defect) and BW312 (BR signaling defect) had a two-fold higher frost tolerance than the wild-type Bowman. The BR biosynthesis mutant 522DK had a several times higher frost tolerance than the wild-type Delisa [94]. 

Climate warming and the subsequent temperature fluctuations in winter are becoming an increasingly serious problem for agriculture in the countries where winter cereals are grown. This fact might create perspectives for cereals with disturbed BR biosynthesis/signaling in breeding programs. This issue will also require further studies for winter cereals other than barley. 

Changes in the tolerance of cereals to temperature stress as a result of manipulating the BR level by both an exogenous application as well as by using mutants with a BR deficiency made it necessary to take a more in-depth look into the endogenous changes of the BR content in plant tissues in relation to temperature especially in relation to the processes of hardening that enable plants to acquire a tolerance to more extreme temperatures.

## 4. Accumulation of Brassinosteroids during the Hardening/Acclimation Processes in Light of the Tolerance of Cereals to Frost or a High Temperature

### 4.1. BR Accumulation in the Leaf Tissue

In cereals, e.g., winter wheat, during cold hardening, the hormonal homeostasis changes significantly, including the hormones from the group of cytokinins, gibberellins, auxins as well as jasmonic acid (JA), salicylic acid (SA), and abscisic acid (ABA) in both the leaves and roots [96]. Similarly, for example, in rice, high-temperature stress alters the hormonal homeostasis, which has various consequences for the yield [97]. In some cases, the phytohormone levels can be correlated with the level of plant tolerance to temperature stress. An increase in the level of ABA occurred in maize as a defensive response to cold stress, while more cold-tolerant cultivars were characterized by a higher content of this hormone [98]. 

Studies related to changes in the level of BR in cereals during cold hardening are rare. In winter rye plants that were grown at 18 °C, the BR castasterone was present in a content of about 4–5 pmol∙g^−1^ F.W. and its concentration increased during the process of cold hardening to about 14–16 pmol∙g^−1^ F.W. [88]. In cultivars of winter wheat, the main BR that was present was homocastasterone (up to 1400 pmol∙g^−1^ F.W.) [89]. Castasterone and teasterone were only detected in amounts of less than 2 pmol∙g^−1^ F.W. As a result of the cold hardening, the BR content increased in the winter wheat leaves [89]. Importantly, more BR (especially homocastasterone and teasterone) accumulated in the wheat cultivars with a higher frost tolerance, while fewer BR were characteristic for the cultivars that were more sensitive to frost [89].

**Figure 3:**
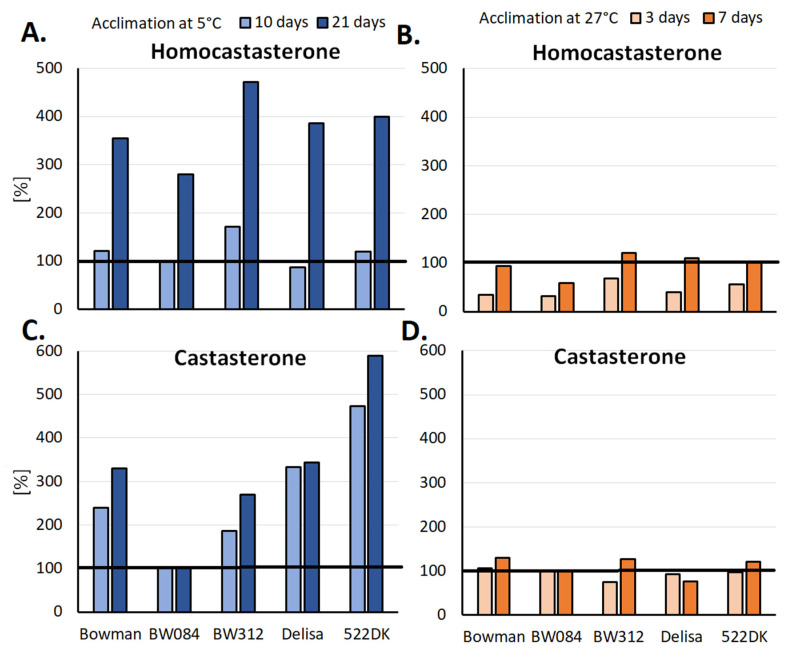
Relative percentage changes in the homocastasterone and castasterone content in the barley plants that had been acclimated at 5 °C (**A**,**C**) and 27 °C (**B**,**D**) relative to plants that had been acclimated at 20 °C. The results that were obtained for the plants that had been grown at 20 °C were considered to be 100% and are indicated by the horizontal black line. Original data of BR accumulation are available in [13]. Bowman—a reference cultivar for two NILs: BW084—plants with disturbances in the early stage of the BR biosynthetic pathway and BW312—plants with a BRI1 receptor defect. Delisa—reference cultivar for the 522DK mutant (plants with disturbances in the late stage of the BR biosynthesis pathway).

Homocastasterone is also a dominant BR in spring barley [13]. During cold hardening (5 °C), all of the tested plants (Bowman, Delisa, BW084, BW312, 522DK) generally accumulated more BR (homocastasterone and also castasterone) compared to the plants that had been grown at 20 °C. In Figure 3A,C, the relative percentage changes in the homocastasterone and castasterone content in the barley plants that had been acclimated at 5 °C relative to those that had been acclimated at 20 °C, respectively, are shown. An increased accumulation of castasterone was already visible in almost all of the tested genotypes (exception: BW084) after ten days of cold hardening. In the case of homocastasterone, the effect was the most intense in all of the genotypes after 21 days. To summarize, the cold-hardened barley accumulated more BR, while the opposite effect was observed in the barley that had been exposed to a higher temperature. As a result of the acclimation of plants at 27 °C, in the initial stage (three days), the homocastasterone content in the barley leaves was lower than in the control plants that had been grown at 20 °C (Figure 3B). After seven days, the content of this hormone returned to its normal level (control). The amount of castasterone was more or less similar in plants at 20 °C and 27 °C.

In the context of the discussion of Figure 3, it should be very clearly emphasized that the original results concerning the BR content that were published in the work of Sadura et al. [13] for the reference cultivars (Bowman and Delisa), the NILs: BW312, BW084, and the 522DK mutant enabled the relationship between the level of BR and the level of tolerance to temperature stress to be observed. The Bowman cultivar, which accumulated the highest amount of homocastasterone, was the most frost tolerant. The NILs BW084 and BW312 as well as the Delisa cultivar and its 522DK mutant did not perform as well as the Bowman in terms of their frost tolerance and they also had lower levels of homocastasterone compared to Bowman. These results are consistent with those that were obtained for winter wheat, where a higher content of BR after cold acclimation was correlated with higher frost tolerance [89]. The opposite effect occurred in the case of tolerance to a high temperature. Both of the NILs, BW084, and BW312, and the 522DK mutant had a lower level of homocastasterone after acclimation at 27 °C, and a higher tolerance to temperature 38 °C and 45 °C than the reference Bowman and Delisa cultivars, respectively [13]. 

The presented results might indicate a special role of homocastasterone in the temperature acclimation of barley (as well as wheat), where its higher level is more favorable for frost tolerance while a lower level (tests in the case of barley) seem to be more beneficial for tolerance to a high temperature. In the case of rye, the only BR that was present was castasterone and its level increased in the cold. However, for this species, the number of tested cultivars was insufficient to unambiguously correlate the level of this hormone with the level of the frost tolerance of the cultivars. 

In conclusion, in cereal crops such as wheat, barley, or rye, BR that do not have an oxalactone ring in the molecule (compare Figure 1) might be important for the development of tolerance to temperature stress. Although the only well-described BRI1 receptor is believed to bind both castasterone and brassinolide equally well [99,100], in some cereals (rice), castasterone, but not brassinolide, is an end product of the BR biosynthetic pathway [101]. No brassinolide was identified in the tested rye and wheat plants. Brassinolide was present in the barley, but interestingly, only at 20 °C. At lower and higher temperatures, its content was below the detection limit, which might indicate that it is only marginally important in barley temperature acclimation [13].

Finally, it is worth mentioning that the defect of the BRI1 receptor in the BW312 plants at both high and low temperatures resulted in an increased accumulation of both BRI1 receptor ligands—castasterone and brassinolide [13]. However, this phenomenon did not apply to homocastasterone—BR was present in the highest amount in both barley and wheat. Therefore, in cereals (barley), can homocastasterone be a ligand of a different BR-binding protein than the only BRI1 receptor that has been characterized to date? Earlier studies by Xu et al. [102] showed the binding of various BRs in both the membrane and cytoplasmic fractions and at the same time the possibility of the presence of different BR-binding proteins and, what is more important, temperature-dependent changes in the accumulation of these proteins (e.g., modified by cold). The more detailed role of homocastasterone, its binding, and participation in the signaling that is associated with temperature stress in cereals will require further research.

### 4.2. Subcellular Location of BR: Chloroplasts

BR biosynthesis enzymes, which belong to the cytochrome P450 family, are located in the endoplasmic reticulum membrane [15,16]. As for the cell fractions, an accumulation of BR was found in wheat and barley chloroplasts [37,103]. The presence of BR in the chloroplasts in some way suggests that they might perform physiological functions such as regulating the transcription of the chloroplast genes [104]. Wheat chloroplasts contain castasterone, homocastasterone, and also 24-epibrassinolide [103]. More BR are accumulated by the wheat cultivar that is more tolerant to oxidative stress than the oxidative stress-sensitive cultivar. The use of exogenous 24-epibrassinolide causes an accumulation of additional amounts of BR from various groups including homocastasterone in the chloroplasts. This phenomenon is more intense in the oxidative stress-tolerant cultivar than in the sensitive cultivar. In turn, eight BRs were found in chloroplasts that had been isolated from barley leaves [37]. In the studies of Sadura et al. [37] in which the BR-deficient mutant 522DK and its reference cultivar Delisa were analyzed, it was observed, among others, that although the level of individual BR (mainly C_28_ brassinolide, castasterone) in the leaf tissues was lower in the mutant than in the wild type, this regularity did not occur in the chloroplasts. In the chloroplasts of the 522DK mutant that had been grown at 20 °C, the content of homocastasterone and 28-norcastasterone was decreased while homodolicholide and homodolichosterone was increased compared to the wild-type chloroplasts. The chloroplasts of the mutant and wild type contained comparable amounts of 24-epibrassinolide, castasterone, brassinolide, and dolicholide. Thus, it can be presumed that there are unknown mechanisms of BR accumulation in the chloroplasts, which results in maintaining the BR level regardless of the general BR level in the tissue (and despite any disturbances in the BR biosynthesis), which especially concerns BR C_28_ because the disturbances in the BR biosynthesis in this group have been clearly confirmed in the tested BR-mutant 522DK [9]. 

In reference to the process of acclimation in the cold and at 27 °C, it is worth noting that the level of individual BR in the chloroplasts also changed [37]; however, in a different way than in the leaves. After acclimation at 5 °C, in the leaves of the 522DK mutant, the content of homocastasterone significantly increased to approximately 7000 pg·g^−1^ F.W., whereas the castasterone level increased to approximately 500 pg·g^−1^ F.W. [13]. There was an opposite tendency in the chloroplasts. The content of castasterone significantly increased to approximately 5000 pg·g^−1^ F.W. and exceeded the content of homocastasterone (1000 pg·g^−1^ F.W.) and the other detected BR [37]. Therefore, it can be suspected that a higher accumulation of castasterone in the chloroplasts, which is the dominant BR in chloroplasts, is more desirable and may be associated with the physiological function that this BR might perform in these organelles during cold acclimation; however, understanding this requires further research (see also Chapter 6.1). In the case of acclimation at 27 °C, the total BR concentration (despite some differences in the level of individual BR) was unchanged (Delisa) or slightly decreased (mutant 522DK) compared to the plants at 20 °C [37], which was a trend that was similar to the observations of leaf tissues [13]. 

## 5. The Effect of Brassinosteroids on Photosynthesis during the Acclimation of Cereals to Low and High Temperatures

### 5.1. Light Reactions of Photosynthesis

The regulation of photosynthesis during the process of cold hardening/acclimation is a well-explored scientific field. Regarding the light reactions of photosynthesis, the efficiency of the most often characterized efficiency of PSII is usually lower in cold. In the winter and spring cultivars of oat (*Avena sativa* L.), four weeks of cold acclimation ((4/2 °C) (d/n)) decreased the values of the maximum quantum efficiency of PSII photochemistry (expressed by F_v_/F_m_ parameter), although over time the effect was more and more reversed, which indicated the adaptive abilities of plants [105]. In barley plants, the maximum quantum efficiency of the PSII photochemistry (F_v_/F_m_ parameter) decreased after 14 days of acclimation at 2 °C [106], and the general PSII efficiency (expressed by P.I._ABS_ index) was also lower in the barley cultivars Bowman and Delisa that had been acclimated at 5 °C (three weeks) [13]. In the other plants from the same cereal family (*Poaceae*), perennial ryegrass, cold hardening (4 °C, three to six weeks) increased the loss of energy as heat (the NPQ parameter) [107]. More information about the reaction of the photosynthetic apparatus in cold-exposed cereals can be also found in the review by Hassan et al. [108].

The application of BR limits the effects of low temperature on the efficiency of the light reactions of photosynthesis, especially when it concerns the energy flow in PSII [88]. In cold-acclimated frost-tolerant winter rye (cultivar Dańkowskie Złote) that had been treated exogenously with 24-epibrassinolide, the energy flow from the photosynthetic antennas to the electron transport chain was more effective than in the untreated control. Simultaneously, the energy that was lost as heat was lower. However, the results that were obtained for the moderately winter-tolerant cultivar Stach revealed a slightly different pattern of changes, BR still decreased the energy losses as heat [88]. Although the positive effects of exogenously applied BR on the photosynthetic light reactions in cereals during cold acclimation were observed, studies on the cereal BR-mutants reveal rather opposite information. BR-barley mutants with defects in BR biosynthesis or signaling (BW084, BW312, 522DK) maintained a higher efficiency of PSII (expressed by the Performance Index P.I._ABS_) than the reference cultivars after three weeks of cold acclimation [13]. This is the opposite effect of what might be expected from studies with the exogenous application of BR. The link between BR biosynthesis and the biosynthesis of chlorophyll in cereals was shown by Liu et al. [109]. The authors noted that the BR-deficient mutant of rice (mutation *lhdd10*) had a higher level of chlorophyll and a higher accumulation of the transcripts of the chlorophyll biosynthetic genes than the wild type. According to the authors, *lhdd10* negatively regulated the expression of the chlorophyll biosynthetic genes, which reduced the chlorophyll content. Considering the fact that in the cold-exposed cultivars of cereals, photosynthetic efficiency decreases [13,105,106], while the level of endogenous BR increases [13,89], it is likely that in natural environmental conditions, one of the functions of BR could be limiting the photosynthetic efficiency during the cold acclimation of winter cereals. Additionally, this thesis might be supported by the results that were obtained in an experiment in which the frost tolerance of BR-mutants of barley was tested [13]. The BR-biosynthetic mutants generally accumulated less BR than the wild-type plants, however, their photosynthetic efficiency was higher, which was accompanied by a lower frost tolerance of the mutants than that of the reference cultivars. To conclude, BR might be negative regulators of the photosynthetic light reactions during cold hardening, which might be a part of the plant acclimation mechanisms that are associated with, i.e., reducing growth in low temperatures. Why, then, in experiments using exogenous BR, was there an improvement in frost tolerance and an accompanying higher PSII efficiency [88]? This is probably because the large amounts of BR that were delivered to the leaf surface also had other beneficial metabolic effects, including improving the stability of the cell membranes in frost [110].

Higher temperatures also affect the functioning of PSII. However, the negative effects are mainly associated with temperatures that significantly exceed the physiological optimum for cereals, for example, 40–45 °C. Temperatures of about 25–30 °C, which can be the basis for acclimation, do not necessarily have such an effect. As was shown for seven genotypes of rice, at temperatures between 20 °C and 27 °C, there were no differences in the maximum quantum efficiency of photosystem II (F_v_/F_m_) [111]. Moreover, no significant differences were observed in the PSII efficiency (expressed as P.I._ABS_) in barley (Bowman and Delisa) at 20 °C or after seven days at 27 °C, while the values of energy absorption by the photosynthetic antennas were even higher at 27 °C (compared to 20 °C) [13]. In rice, the tolerance of temperatures between 27 °C and 37 °C was more genotype dependent—the values of F_v_/F_m_ varied more strongly between the genotypes at 37 °C compared to 20 °C [111]. The growth of winter wheat cultivars at temperatures of 35/20 °C d/n (15 days) caused a gradual decrease in the PSII effective quantum yield (ΦPSII), especially from the 11th day of stress [112]. Treatment with more extreme temperatures (40 °C) rapidly reduced the values of F_v_/F_m_ in rice by half and finally at 50 °C to zero [111]. Other studies on rice also confirmed that temperatures of 40/30 °C (d/n) caused a gradual decrease in the value of the maximum quantum efficiency of the PSII photochemistry (F_v_/F_m_ parameter) [79,91]. A decrease in PSII efficiency was also observed for a few cultivars of barley that had been grown at temperatures higher than 40 °C [13]. Although the protective effect of exogenous BR on the efficiency of the light reactions of light photosynthesis should also be observed in this context, it was only described for more extreme temperature treatments of around 40 °C, e.g., in barley and rice [79,90,91]. A better performance of the photosynthetic light reactions (mainly within PSII) was usually accompanied by a higher chlorophyll content that was caused by the application of BR [79,91]. 

The positive effect of exogenously applied BR on the photosynthetic efficiency of heat-exposed cereals could lead to the conclusion that disturbances in BR biosynthesis or defects in BR signaling would result in a worse efficiency of the light reactions of the photosynthesis of BR mutants at higher temperatures. Surprisingly, in barley BR mutants or near-isogenic lines (NILs) with the aforementioned defects, the efficiency of the light reactions of photosynthesis was better than in wild types not only at 20 °C or 27 °C but also after exposure to 38 °C and 45 °C [13]. One explanation for this phenomenon might be that BR-mutants have darker green leaves, which affects the photosynthetic measurements using fluorescence methods [37]. A second likely cause might be that high-temperature stress is often accompanied by the drought stress that results from a significantly intensified transpiration. Plants with a disturbed BR biosynthesis/perception are semi-dwarf and have a smaller leaf area than the wild-type cultivars and therefore they lose less water when exposed to high temperatures. 

Unlike the cold-induced changes in the efficiency of the light reactions of photosynthesis (and the BR level), the acclimation of cereals to higher temperatures (understood as treatment of plants with temperatures of around 27–30 °C) is not associated with significant changes in the efficiency of the light reactions of photosynthesis compared to 20 °C. That is why the role of BR also seems to be less important in this context. Its role might increase when plants are exposed to more extreme temperatures of around 40 °C. Interestingly, however, both plants that had been treated with exogenous BR (with a higher level of BR) and the BR-defective mutants exhibited higher tolerance to a high temperature, which in both cases was often accompanied by a higher chlorophyll accumulation and a higher PSII efficiency. Thus, the mechanisms of BR action must be different in these two models of studies but they lead to the same effect. 

### 5.2. Dark Reactions of Photosynthesis and Sugar Accumulation

Regarding the dark reactions of photosynthesis, after six weeks of the cold acclimation of winter rye and perennial ryegrass, the activity of the CO_2_-binding enzyme (Rubisco) was higher than before cold acclimation [88,107]. The increase in the Rubisco activity was more or less associated with the increased need for sugars. The phenomenon of the higher accumulation of soluble sugars, including glucose, fructose, and especially sucrose during cold exposition is very well known [113]. A higher sucrose concentration reduces the freezing point of a cell aqueous solution; thus, it generally contributes to a higher tolerance to low temperatures. This phenomenon has been observed as the mechanism of the cold response for a few winter species such as rye [88] or wheat [114], in grass—perennial ryegrass [107], and even in cold-sensitive maize [87]. As was described in the chapter about the light reactions of photosynthesis, the results of experiments with the exogenous treatments of BR and BR-deficient mutants were not very compatible. By contrast, the results of the studies of the impact of BR on the Rubisco activity or sugar accumulation in plants that had been treated with BR or mutants/transgenic plants were more compatible. Treating winter rye with BR (24-epibrassinolide), together with the plants’ exposure to cold, stimulated the Rubisco activity and the accumulation of soluble sugars, although some cultivar dependency was observed [88]. The effect of cold on sugar management was also described for maize (inbred line LM-17) by Singh et al. [87]. Seedlings that had been exposed to cold stress (in a net house) exhibited a slight increase in the amount of glucose, starch, and also sucrose compared to the control plants that had been cultured in the controlled conditions of a greenhouse. The application of 24-epibrassinolide (1μM) also increased the concentrations of glucose, starch, and sucrose by about 15%, 45%, and 28%, respectively, compared to the stressed maize without the BR treatment. 

An increase in the content of BR can also be achieved in transgenic cereals. In transgenic rice, expressing maize, rice, or the *Arabidopsis thaliana* genes encoding sterol C-22 hydroxylases, which control the BR level, the concentration of, among others, 6-deoxocatasterone, 3-epi-6-deoxocatasterone, 6-deoxoteasterone, 6-deoxotyphasterol and typhasterol was higher in leaves [115]. A microarray and photosynthetic analysis showed an increase in CO_2_ assimilation together with enlarged glucose pools in the flag leaves as well as an increase in the assimilation of glucose to starch in the seeds. According to the authors, BR might not only stimulate synthesis but also the flow of the assimilates. Simultaneously, the BR-deficient mutants 522DK and 527DK with a lower BR content, which were grown in optimal conditions, were characterized by a lower Rubisco activity and a lower accumulation of sucrose, which was accompanied by a higher accumulation of fructose and glucose [116]. 

High temperatures also restrict the dark reactions of photosynthesis [117,118,119]. However, studies of the role of BR in alleviating the negative effects of high temperatures on the dark reactions of photosynthesis are rather concentrated on the effects of extreme temperatures not on the acclimation processes. Unlike low temperatures, a high temperature (40 °C) caused only a slight decrease in the Rubisco activity (by about 10%) in cereals (wheat) compared to the control, which had not been subjected to a high temperature [120]. 24-Epibrassinolide reduced this effect and as a result the total Rubisco activity was higher compared to the BR-untreated plants [120]. 24-Epibrassinolide was also active in reducing the negative effects of high temperature on the dark reactions of photosynthesis in rice [79,91]. After heat stress, the plants that had been pretreated with BR had higher values of net photosynthesis (P_N_) compared to the untreated and heat-exposed plants of the control [79,91]. This was probably one of the factors that also contributed to the increased accumulation of total soluble sugars in the BR-pretreated and heat-exposed plants [91]. A marked decrease in the total soluble sugar content was found in rice plants (cv. Pathum Thani 1) that had been subjected to heat stress (40/30 °C; seven days) compared to the plants that had not been exposed to a high temperature [91]. An increase in the total soluble sugar content (by about 23%) was recorded in the plants that had been pretreated with 24-epibrassinolide compared to the plants that had been exposed to heat without BR being applied. In the same cultivar, which was grown in field conditions during the hot season (February to May 2012, Thailand), 24-epibrassinolide increased the total soluble sugar content in the straw (by 107%), husk (by 5%), and seeds (by 8%) [79]. In other cultivars of rice—IR-64 and Huanghuazhan—that had been exposed to temperatures of 35/32 °C (d/n) for 12/12 h, there was a decrease in the soluble sugar content, which was significantly reduced by a mixture of plant growth regulators containing BR [92].

To summarize, when it comes to the widely understood process of photosynthesis and its final effects (production of carbohydrates), it can be concluded that in the case of cold acclimation (hardening), BR has a positive effect that is mainly associated with the accumulation of soluble sugars. This is a well-known and important phenomenon of adaptation to this stress and—what is particularly important in the case of winter cereals—it improves frost tolerance. High temperatures essentially reduce the light reactions of photosynthesis while at the same time, the dark respiration processes are intensified, which can finally result in reducing the sugar resources. The action of BR in plants is manifested by smaller losses of sugars, which enables them to preserve their resources for other metabolic processes as well as for growth.

## 6. The Effect of Brassinosteroids on the Cell Membrane—Its Importance for the Process of Acclimation to Low and High Temperatures in Cereals

### 6.1. Membrane Lipids

Cell membranes play an important role in the plant-developed strategies for dealing with the negative effects of abiotic stresses, including the effects of temperature stress [121]. According to Horváth et al. [122], cell membranes can be considered to be “thermal sensors” and modifications in membranes may be a prelude to many metabolic changes in cells, including the expression of specific genes. During acclimation to lower or higher temperatures, the changes in the fluidity of the cell membranes are associated with a modification in the proportion of unsaturated/saturated fatty acids, which results in a rearrangement of the membrane structure and in a change in its properties [47]. Typically, during cold hardening, the incorporation of unsaturated fatty acids is beneficial for the functioning of cellular membranes at a low temperature, while the reverse phenomenon (more saturated fatty acids) is associated with acclimation to a higher temperature [110,123,124]. The properties of membranes might also be modified as a result of incorporating various components into them: sterols, tocopherols, and carbohydrates [75,125]. When investigating the potential role of BR in modifying the properties of the cell membranes (as part of the acclimation process to low or high temperatures), it is well-founded to focus on the possible effect of BR on the biosynthesis of fatty acids (FA) or the possibility of BR being incorporated into the membranes. 

Barley with defects in BR-biosynthesis and BR-signaling is characterized by a temperature-dependent altered molar percentage of fatty acids (from 14:0 to 20:1) in their phospholipid and galactolipid fractions compared to the reference cultivar [36]. Specifically, the mutants had a lower molar percentage of 18:3 FA in their phospholipid fraction and this regularity was maintained at 5 °C while the reverse effect was observed at 27 °C. In barley, the phospholipid fraction is the main fraction that is present in the plasmalemma [126]. On the other hand, unsaturated FA 18:3 in barley is the main FA in the phospholipid fraction (about 50%) [36]. The content of 18:3 in this fraction increased naturally during the acclimation to a low temperature (up to 56%), while it decreased during plant growth at 27 °C (to 46%) in the barley cultivar Bowman [36], which is connected to an adjustment of the cell membrane fluidity (higher or lower, respectively) to various temperature conditions. As was mentioned earlier, in barley with BR biosynthesis and signaling defects at a temperature of 5 °C, the molar percentage of this FA was lower while it was higher at 27 °C, which correlated well with the fluidity of the membrane, which is expressed as the limiting area per molecule (A_lim_)—measured in Langmuir bath studies. The mutations are connected with lower values of A_lim_ at 5 °C, while there is a higher A_lim_ at 27 °C [36]. Because the plasmalemma is important in the process of acclimation to temperature, this direction of changes could be one of the many reasons for the lower tolerance of the mutants to frost and a higher tolerance to heat stress [13]. On the other hand, a reverse effect is observed in the case of the monogalactolipid fraction (more characteristic for the chloroplast membranes [127,128]) compared to the phospholipid fraction. In the BR mutants at 5 °C, 18:3 was higher (and fluidity as well) while at 27 °C (and 20 °C), it was lower (together with a decreased fluidity, which was expressed by A_lim_). Such a phenomenon in the chloroplast membranes might provide better conditions for the functioning of the photosystems (and the light reactions of photosynthesis) at 5 °C and 27 °C [13]. Generally, however, we must remember that the cell membranes are composed of various proportions of the fractions that have been mentioned and that their physicochemical properties will be the result of this mosaic system and therefore, the results of studies on pure fractions should be treated with some caution. Moreover, it can be assumed that BR either participate in the temperature-dependent regulation of FA biosynthesis or control the processes that are responsible for the transport or incorporation of FA into the membranes. The issue requires further detailed studies that are devoted to the mechanisms of the BR-induced changes in the lipid composition. 

As was mentioned earlier, the membrane properties can be modified via the incorporation of various compounds into the membranes. Sterols are especially known to be such modifiers in both animals and plants [129]. Brassinosteroids have a structure that is similar to that of sterols and in order to describe the BR-lipid membrane interactions, model studies have been conducted using the Langmuir technique [110]. Membrane lipids have been isolated from winter wheat plants (control and cold hardened) with varying frost tolerances. The monolayers of phospholipids and monogalactolipids, which were isolated from the cultivar that was the most frost tolerant (Smuga) after cold acclimation (5 °C), were characterized by the highest fluidity compared to the moderately tolerant Nutka and the sensitive Bystra. When BR (24-epibrassinolide and 24-epicastasterone) were added to the lipid fractions, changes in the fluidity of those monolayers were observed. The interaction of specific BR (24-epibrassinolide/24-epicastasterone) with a specific monolayer (phospholipids and monogalactolipids) was slightly different. 24-Epibrassinolide increased the fluidity of the monolayers of the phospholipids while 24-epicastasterone decreased it. 24-Epibrassinolide only slightly increased the fluidity of the monolayers from the monogalactolipids while 24-epicastasterone clearly increased it. Differences in the BR structure (the presence of an oxygen bridge in the B-ring of 24-epibrassinolide, but not in 24-epicastasterone) might be responsible for the differently modified structure of the monolayers and their properties. The higher polarity of BR (24-epibrassinolide) probably favors its placement closer to the polar part of the membrane and the formation of the so-called liquid-disordered ‘rafts’ in the phospholipid-containing membranes. On the other hand, the BR that are characterized by a lower polarity (24-epicastasterone) could be closer to the hydrophobic part of the membrane, thereby stabilizing the monolayer in the more liquid-ordered phases. At this point, it is worth mentioning that the studies of Janeczko et al. [89] showed that when applied exogenously to the leaf surface of winter wheat, 24-epibrassinolide increased the tolerance of plants to frost while castasterone decreased it. It is likely that one of the mechanisms here might be the different effect that these two compounds have on membrane fluidity (more unequivocal in the case of 24-epibrassinolide). 

Because the influence of BR on the physicochemical properties of the monolayers has been observed in model studies [110], in the next step, natural chloroplast membranes were used to verify the effect of BR on their molecular dynamics [37]. First of all, it was established that BR are present in the chloroplasts of wheat [103] and barley [37]. In the case of the barley chloroplasts, BR were detected in four-fold higher amounts than in the leaves [13]. The temperature-dependent accumulation of mostly large amounts of BR in the chloroplasts was an indication that these compounds might perform physiological functions in these organelles including regulating membrane fluidity. The most abundant BR in the chloroplasts was castasterone [37]. In the case of the cultivar Delisa, its level was almost tripled after cold acclimation while it was slightly lower after acclimation to 27 °C (compared to the chloroplasts at 20 °C). Since it was previously noted that a similar compound (24-epicastasterone) increased the fluidity of the monogalactolipid monolayers [110] (and galactolipids are the main component of the chloroplast membranes [130], an increase in the level of castasterone in cold led us to suppose that one of the functions of this hormone could be regulating the fluidity of the chloroplast membranes. 

Interestingly, despite the decreased level of BR (castasterone) in the leaf tissue of the BR-deficient barley mutant (522DK) [13], the concentration of this hormone in the chloroplasts remained at the level that was observed in the plants of the reference cultivar Delisa [37]. The mechanisms that are responsible for the accumulation of BR in the chloroplasts of cereals are currently unknown. How this mechanism works can only be hypothesized by paying attention to the steroid hormones that are found in animals such as progesterone, testosterone, or estradiol. Due to their hydrophobicity, these hormones are able to freely diffuse across the membranes in animal cells [131]. At the same time, different functional groups of these hormones affect their affinity for the lipid bilayer as well as the rate of their diffusion through the membrane. The presence of polar groups in the tail (side chain) of a steroid in animals is believed to introduce a barrier to this free diffusion through the hydrophobic core of a membrane [132]. It can be assumed that it might look similar in the case of BR, which are polyhydroxysteroids. Depending on the number of hydroxyl groups and their presence in the side chain, these compounds can be incorporated into a membrane or, if there are fewer polar groups, the BR can be diffused. Thus, the highest content of castasterone in the chloroplast membranes might result from the lower hydrophobicity of its structure compared to, for example, homocastasterone, which additionally has an ethyl group in the C24 position. Homocastasterone was also detected in chloroplasts, but interestingly, in the leaves, it was present in a much larger amount than castasterone [13,37]. Therefore, homocastasterone is allowed to remain outside of the chloroplasts to a greater extent while castasterone has a greater ability to pass through a membrane. The chemical composition of the lipid bilayer may also play an important role as it may favor, for example, a faster diffusion or inhibit it. 

**Figure 4:**
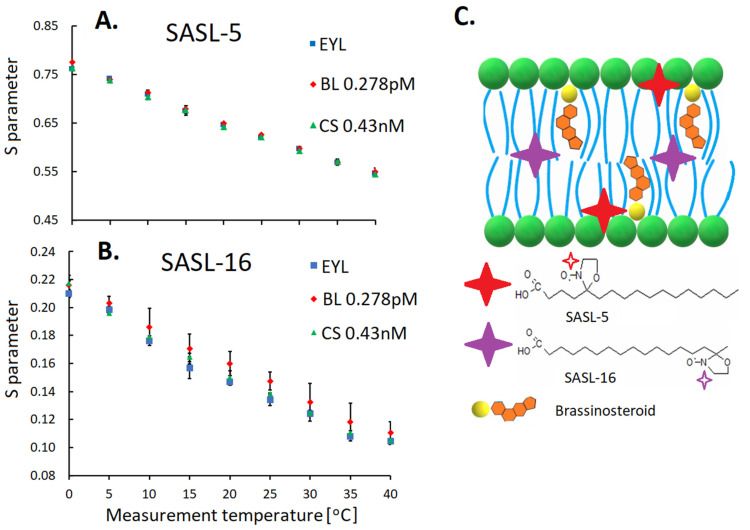
Dependence of the order parameter S of the model membranes that had been prepared from egg yolk lecithin (EYL) with incorporated spin labels SASL-5 (**A**) or SASL-16 (**B**) and BR (BL—brassinolide; CS—castasterone at sample concentrations corresponding to their natural concentrations in chloroplasts) on the measurement temperature of the spectra using the EPR method. (**C**) Model of the prepared model membrane. The liposomes were prepared according to the modified method described earlier in work Sadura [135]. Briefly, spin labels (SASL-5 or SASL-16) (0.1 mM) and BR (BL or CS) (at a certain concentration) were added to EYL (10 mM). Next, it was shaken on a vortex and centrifuged and the methanol (in which all ingredients were suspended) was evaporated under N_2_ in order to obtain a film on the test tube walls. After that, a CIB buffer (pH 7.5) (described in Sadura et al. [37]) was added to the sample and it was shaken for one minute on a vortex. Next, the sample was placed in a capillary, and the EPR spectra were measured in a temperature range of 0 °C to 40 °C at intervals of 5 °C (the process of the measurements is described in Sadura et al. [37]). The presented dependence enabled a conclusion to be drawn about the molecular dynamics of the membranes. Order parameter S was calculated according to the equations presented in the work of Sadura et al. [37]. The graphs show the mean values of two measurements ± SD.

To verify the results of earlier studies, which were conducted using a Langmuir bath regarding the possibility that BR have an impact on the molecular dynamics of membranes, electronic paramagnetic resonance (EPR) was used to compare the molecular dynamics of the chloroplast membranes that had been isolated from a barley mutant with a disturbed biosynthesis of BR (522DK) and one that had been isolated from its reference cultivar Delisa (plants were growing at 20 °C and at 5 °C and 27 °C) [37]. The EPR method consists in incorporating the so-called spin labels/spin probes, which are molecules with permanent paramagnetic centers, mostly in the form of the nitroxyl groups. Depending on where (e.g., at which carbon atom) the spin label has its paramagnetic center, after its incorporation into a membrane, it is possible to draw a conclusion about its molecular dynamics at a given depth. To date, in this kind of study, exogenous BR were used, for example, in the case of mango fruits [133]. The molecular dynamics of barley chloroplast membranes is naturally dependent on temperature [37]. However, only slight differences in the molecular dynamics were obtained between the 522DK mutant and the reference cultivar, which had most likely resulted from the comparable total BR content (and the content of the most abundant castasterone) in the mutant and the reference cultivar [37]. That is why in the next step, studies in which the BR were incorporated into the membranes (prepared from egg yolk lecithin) at various physiological concentrations, which were calculated based on the BR content in the chloroplasts (0.278 pM of brassinolide and 0.43 nM of castasterone) and at higher concentrations of up to 0.1 mM of each BR were also performed. However, the studies also did not permit the effect of BR at such concentrations to be proven on the molecular dynamics of the studied membranes (Sadura and Latowski, unpublished data; Figure 4). In the next studies, membranes with a composition of fatty acids that were more similar to the natural membranes that are present in plant cells (containing 18:3 FA) were used, and once again higher concentrations of BR were tested [4:1 (lipids: BR)] [134]. In the case of such a high proportion of lipids vs. BR, it was again confirmed that the differences in the chemical structure of the BR (homocastasterone and castasterone) are an important factor in the incorporation of BR and their placement in the membrane structure. BR also affected the properties of these membranes (molecular dynamics—EPR studies, membrane fluidity—Langmuir bath technique). 

To summarize, BR were found in the chloroplasts (chloroplast membranes), and their level changed as a result of the acclimation to low and high temperatures. The amount of the main BR (castasterone) increased at 5 °C and was lower at 20 °C and 27 °C. The molecular dynamics of the membranes (measured using EPR) was however similar in both the mutant with a disturbed BR biosynthesis and in the reference cultivar. The administration of BR in the physiological amounts (characteristic for chloroplasts) did not change the molecular dynamics of the model membranes from egg yolk lecithin. Both the molecular dynamics of the membranes and their physicochemical properties that were determined in the studies with the Langmuir bath changed after using BR but at higher concentrations (1:4—BR:lipid). This may suggest a local effect of BR in the cellular membrane in the so-called ‘rafts’, where hormones would be concentrated in higher amounts. This is more likely as the amount of BR in the chloroplast membranes compared to, e.g., the content of sterols therein, was small. The issue requires further detailed studies.

### 6.2. Membrane Proteins

Membrane proteins play a role in membrane functioning and are important elements in dealing with temperature stress. Examples of these proteins are the heat shock proteins (HSP), the water transporting proteins (aquaporins), and the proton pump (H^+^-ATPase). HSP are characterized by their different molecular weights, namely, HSP100, HSP90, HSP70, HSP60, and small heat-shock proteins (sHSP). The main role of HSP is to act as the molecular chaperones of other proteins, and although their name derives from the fact that they were found in plants that had been exposed to high temperatures, these proteins are commonly found in plants that have been grown at both “room temperature” and in cold conditions. They are responsible, among others, for regulating protein folding as well as its accumulation, location, translocation, and degradation in all plant and animal species [136,137]. Aquaporins are transmembrane proteins, which were discovered as the water channels that transport water through the cell membranes. The main function of the plasma membrane intrinsic proteins (PIP) in plants is to regulate the transmembrane water transport when the water flow needs to be modified or when it is very low [138]. Attention should be paid to this protein in terms of adapting plants to different temperatures because the dehydration and hydration of cells are crucial in both adapting to frost and surviving high temperatures by a plant [139,140,141]. H^+^-ATPase is a membrane-bound protein that is called a membrane proton pump, which is responsible for the active transport of cations or other compounds across the membranes and this process is coupled with ATP hydrolysis and, consequently, for generating the proton motor force and driving the secondary transport processes [142]. Generally, in barley, the accumulation of the HSP, PIP, and ATPase transcripts is dependent on the temperature of growth. The cold acclimation of barley (21 days) increased the accumulation of transcript HSP70 and decreased ATPase and HvPIP, while HSP90 was unchanged and sHSPs were not detectable compared to the plants at 20 °C [34,35]. In the barley cultivars that had been acclimated at 27 °C (seven days), the levels of the HSP70 and ATPase proteins were unchanged, HvPIP and sHSPs increased, and HSP90 decreased. Barley with defects in the BR biosynthesis or signaling generally had a lower level of these transcripts, which suggests that these hormones are positive regulators of the transcription of HSP, ATPase, or aquaporins (which is not dependent on temperature). However, the accumulation of these proteins in the BR mutants was different, which indicates that the control of their biosynthesis under various temperature conditions is more complex and is not only dependent on the cultivar or BR but also probably on many other factors [34,35].

## 7. Conclusions

The directions of brassinosteroid activity that are described in this paper suggest that BR are one of the players, in addition to gibberellins or ABA, that are important in acquiring a tolerance to a high temperature and especially to frost in cereals. The influence of BR on increasing the production of sugars or increasing the fluidity of the membranes is certainly a significant contribution of these hormones to the acquisition of tolerance to a low temperature. However, more in-depth, genetic studies are also required to explain the molecular mechanism of the effect of BR on sugar and fatty acid metabolism. Endogenous BR (mainly homocastasterone or castasterone) are accumulated in cereal tissues during the cold-hardening process. The cultivars that are tolerant to frost are characterized by a higher accumulation of BR (mainly homocastasterone, in some cases, also castasterone or teasterone) during cold hardening/acclimation than those that are susceptible to frost. This might suggest that in cereal crops such as wheat, barley, or rye, the BR that do not have an oxalactone ring in the molecule might be important for developing a tolerance to temperature stress. Interestingly, however, in the case of the exogenous application of BR, BR with oxalactone rings in the molecule (24-epibrassinolide) are more effective protectants. This might be explained by other mechanisms of the action of the endogenous and exogenous BR, and this matter requires further in-depth research. Nevertheless, the protective activity of the selected exogenous BR against more extreme temperatures offers the possibility of using them as special agrochemicals in order to improve the tolerance of cereals to temperature stress.

## Data Availability

Not applicable.

## References

[1] Grove M.D., Spencer G.F., Rohwedder W.K., Mandava N., Worley J.F., Warthen J.D., Steffens G.L., Flippen-Anderson J.L., Cook J.C. (1979). Brassinolide, a plant growth-promoting steroid isolated from Brassica napus pollen. Nature.

[2] Liu J., Zhang D., Sun X., Ding T., Lei B., Zhang C. (2017). Structure-activity relationship of brassinosteroids and their agricultural practical usages. Steroids.

[3] Bajguz A., Tretyn A. (2003). The chemical characteristic and distribution of brassinosteroids in plants. Phytochemistry.

[4] Bishop G.J. (2003). Brassinosteroid mutants of crops. J. Plant Growth Regul..

[5] Sadura I., Janeczko A. (2018). Physiological and molecular mechanisms of brassinosteroid-induced tolerance to high and low temperature in plants. Biol. Plant..

[6] Yokota T., Ogino Y., Takahashi N., Saimoto H., Fujioka S., Sakurai A. (1990). Brassinolide is biosynthesized from castasterone in catharanthus roseus crown gall cells. Agric. Biol. Chem..

[7] Fujioka S., Yokota T. (2003). Biosynthesis and metabolism of brassinosteroids. Annu. Rev. Plant Biol..

[8] Clouse S.D. (2015). A history of brassinosteroid research from 1970 through 2005: Thirty-five years of phytochemistry, physiology, genes, and mutants. J. Plant Growth Regul..

[9] Gruszka D., Szarejko I., Maluszynski M. (2011). Identification of barley DWARF gene involved in brassinosteroid synthesis. Plant Growth Regul..

[10] Dockter C., Gruszka D., Braumann I., Druka A., Druka I., Franckowiak J., Gough S.P., Janeczko A., Kurowska M., Lundqvist J. (2014). Induced variations in brassinosteroid genes define barley height and sturdiness, and expand the green revolution genetic toolkit. Plant Physiol..

[11] Altmann T. (1999). Molecular physiology of brassinosteroids revealed by the analysis of mutants. Planta.

[12] Bishop G.J., Yokota T. (2001). Plants steroid hormones, brassinosteroids: Current highlights of molecular aspects on their synthesis/metabolism, transport, perception and response. Plant Cell Physiol..

[13] Sadura I., Pociecha E., Dziurka M., Oklestkova J., Novak O., Gruszka D., Janeczko A. (2019). Mutations in the HvDWARF, HvCPD and HvBRI1 genes-involved in brassinosteroid biosynthesis/signalling: Altered photosynthetic efficiency, hormonal homeostasis and tolerance to high/low temperatures in barley. J. Plant Growth Regul..

[14] Gruszka D., Gorniak M., Glodowska E., Wierus E., Oklestkova J., Janeczko A., Maluszynski M., Szarejko I. (2016). A reverse-genetics mutational analysis of the barley HvDWARF gene results in identification of a series of alleles and mutants with short stature of various degree and disturbance in BR biosynthesis allowing a new insight into the process. Int. J. Mol. Sci..

[15] Jørgensen K., Rasmussen A.V., Morant M., Nielsen A.H., Bjarnholt N., Zagrobelny M., Bak S., Møller B.L. (2005). Metabolon formation and metabolic channeling in the biosynthesis of plant natural products. Curr. Opin. Plant Biol..

[16] Gruszka D., Małuszyński M. (2010). Brasinosteroidy struktura chemiczna, genetyczne podstawy biosyntezy i transdukcji sygnału oraz funkcje fizjologiczne. Postępy Biologii Komórki.

[17] Wang Z.Y., Wang Q., Chong K., Wang F., Wang L., Bai M., Jia C. (2006). The brassinosteroid signal transduction pathway. Cell Res..

[18] Wang W., Bai M.Y., Wang Z.Y. (2014). The brassinosteroid signaling network-a paradigm of signal integration. Curr. Opin. Plant Biol..

[19] Li J., Jin H. (2007). Regulation of brassinosteroid signaling. Trends Plant Sci..

[20] Chung Y., Choe S. (2013). The regulation of brassinosteroid biosynthesis in Arabidopsis. Crit. Rev. Plant Sci..

[21] Planas-Riverola A., Gupta A., Betegon-Putze I., Bosch N., Ibanes M., Cano-Delgado A. (2019). Brassinosteroid signaling in plant development and adaptation to stress. Development.

[22] Yang C.J., Zhang C., Lu Y.N., Jin J.Q., Wang X.L. (2011). The mechanisms of brassinosteroids’ action: From signal transduction to plant development. Mol. Plant.

[23] Holá D., Hayat S., Ahmad A. (2011). Brassinosteroids and photosynthesis. Brassinosteroids: A Class of Plant Hormone.

[24] Mazorra L.M., Hayat S., Ahmad A. (2011). Brassinosteroid action and its relation with heat stress mechanisms in plants. Brassinosteroids: A Class of Plant Hormone.

[25] Nolan T.M., Vukašinović N., Liu D., Russinova E., Yin Y. (2020). Brassinosteroids: Multidimensional regulators of plant growth. Plant Cell.

[26] Fariduddin Q., Yusuf M., Ahmad I., Ahmad A. (2014). Brassinosteroids and their role in response of plants to abiotic stresses. Biol. Plant..

[27] Kothari A., Lachowiec J. (2021). Roles of brassinosteroids in mitigating heat stress damage in cereal crops. Int. J. Mol. Sci..

[28] Li S., Zheng H., Lin L., Wang F., Sui N. (2021). Roles of brassinosteroids in plant growth and abiotic stress response. Plant Growth Regul..

[29] Janeczko A., Hayat S., Yusuf M., Bhardwaj R., Bajguz A. (2019). Brassinosteroids in cereals—Presence, physiological activity and practical aspects. Brassinosteroids: Plant Growth and Development.

[30] Mazorra L.M., Holton N., Bishop G.J., Núñez M. (2011). Heat shock response in tomato brassinosteroid mutants indicates that thermotolerance is independent of brassinosteroid homeostasis. Plant Physiol. Biochem..

[31] Setsungnern A., Muñoz P., Pérez-Llorca M., Müller M., Thiravetyan P., Munné-Bosch S. (2020). A defect in BRI1-EMS-SUPPRESSOR 1 (bes1)-mediated brassinosteroid signaling increases photoinhibition and photo-oxidative stress during heat stress in Arabidopsis. Plant Sci..

[32] Eremina M., Rozhon W., Poppenberger B. (2016). Hormonal control of cold stress responses in plants. Cell. Mol. Life Sci..

[33] Fang P., Yan M., Chi C., Wang M., Zhou Y., Zhou J., Shi K., Xia X., Foyer C.H., Yu J. (2019). Brassinosteroids act as a positive regulator of photoprotection in response to chilling stress. Plant Physiol..

[34] Sadura I., Libik-Konieczny M., Jurczyk B., Gruszka D., Janeczko A. (2020). Plasma membrane ATPase and the aquaporin HvPIP1 in barley brassinosteroid mutants acclimated to high and low temperature. J. Plant Physiol..

[35] Sadura I., Libik-Konieczny M., Jurczyk B., Gruszka D., Janeczko A. (2020). HSP transcript and protein accumulation in brassinosteroid barley mutants acclimated to low and high temperatures. Int. J. Mol. Sci..

[36] Rudolphi-Szydło E., Sadura I., Filek M., Gruszka D., Janeczko A. (2020). The impact of mutations in the HvCPD and HvBRI1 genes on the physicochemical properties of the membranes from barley acclimated to low/ high temperatures. Cells.

[37] Sadura I., Latowski D., Oklestkova J., Gruszka D., Chyc M., Janeczko A. (2021). Molecular dynamics of chloroplast membranes isolated from wild-type barley and a brassinosteroid-deficient mutant acclimated to low and high temperatures. Biomolecules.

[38] Végh B., Marček T., Karsai I., Janda T. (2018). Darkó Heat acclimation of photosynthesis in wheat genotypes of different origin. S. Afr. J. Bot..

[39] Hüner N.P.A., Dahal K., Bode R., Kurepin L.V., Ivanov A.G. (2016). Photosynthetic acclimation, vernalization, crop productivity and ‘the grand design of photosynthesis’. J. Plant Physiol..

[40] Dahal K., Kane K., Gadapati W., Webb E., Savitch L.V., Singh J., Sharma P., Sarhan F., Longstaffe F.J., Grodzinski B. (2012). The effects of phenotypic plasticity on photosynthetic performance in winter rye, winter wheat and Brassica napus. Physiol. Plant..

[41] Janda T., Tajti J., Hamow K., Marček T., Ivanovska B., Szalai G., Pál M., Zalewska E.D., Darkó É. (2021). Acclimation of photosynthetic processes and metabolic responses to elevated temperatures in cereals. Physiol. Plant..

[42] Dalmannsdottir S., Jørgensen M., Rapacz M., Østrem L., Larsen A., Rødven R., Rognli O.A. (2017). Cold acclimation in warmer extended autumns impairs freezing tolerance of perennial ryegrass (Lolium perenne) and timothy (Phleum pratense). Physiol. Plant..

[43] Fürtauer L., Weiszmann J., Weckwerth W., Nägele T. (2019). Dynamics of plant metabolism during cold acclimation. Int. J. Mol. Sci..

[44] Hasanfard A., Rastgoo M., Izadi Darbandi E., Nezami A., Chauhan B.S. (2021). Regeneration capacity after exposure to freezing in wild oat (Avena ludoviciana Durieu.) and turnipweed (Rapistrum rugosum (L.) All.) in comparison with winter wheat. Environ. Exp. Bot..

[45] Darkó É., Khalil R., Elsayed N., Pál M., Hamow K.Á., Szalai G., Tajti J., Nguyen Q.T., Nguyen N.T., Le V.N. (2019). Factors playing role in heat acclimation processes in barley and oat plants. Photosynthetica.

[46] Takahashi D., Li B., Nakayama T., Kawamura Y., Uemura M. (2013). Plant plasma membrane proteomics for improving cold tolerance. Front. Plant Sci..

[47] Niu Y., Xiang Y. (2018). An overview of biomembrane functions in plant responses to high-temperature stress. Front. Plant Sci..

[48] Guo X., Liu D., Chong K. (2018). Cold signaling in plants: Insights into mechanisms and regulation. J. Integr. Plant Biol..

[49] Bohn M., Lüthje S., Sperling P., Heinz E., Dörffling K. (2007). Plasma membrane lipid alterations induced by cold acclimation and abscisic acid treatment of winter wheat seedlings differing in frost resistance. J. Plant Physiol..

[50] Koyro H.-W., Ahmad P., Geissler N., Ahmad P., Prasad M. (2012). Abiotic stress responses in plants: An overview. Environmental Adaptations and Stress Tolerance of Plants in the Era of Climate Change.

[51] Hasanuzzaman M., Hossain M.A., da Silva J.A.T., Fujita M., Venkateswarlu B., Shanker A., Shanker C., Maheswari M. (2012). Plant response and tolerance to abiotic oxidative stress: Antioxidant defense is a key factor. Crop Stress and Its Management: Perspectives and Strategies.

[52] Łączyński A. (2020). Wstępna ocena przezimowania upraw w 2020 r. (Preliminary assessment of the crops wintering in 2020.). Stat. Pol..

[53] Baruth B., Bassu S., Bussay A., Ceglar A., Cerrani I., Chemin Y., De Palma P., Fumagalli D., Lecerf R., Manfron G. (2020). Crop monitoring in Europe. JRC MARS Bull..

[54] Baruth B., Bettio M., Chuckaliev O., Bojanowski J., Bussay A., Duveiller G., Fontana G., Kasperska-Wolowicz W., Lopez R., Maiorano L. (2012). Crop monitoring in Europe. MARS Bull..

[55] Popkiewicz M. Nauka o klimacie (Climate Science). https://naukaoklimacie.pl/aktualnosci/coraz-czestsze-susze-w-polsce-konsekwencja-zmiany-klimatu-i-dzialan-anty-adaptacyjnych-417.

[56] Selin H., Mann M.E. Global Warming. Earth Science. https://www.britannica.com/science/global-warming.

[57] Bradford A., Pappas S. Effects of Global Warming. https://www.livescience.com/37057-global-warming-effects.html.

[58] Kaca E., Labedzki L., Lubbe I. (2011). Gospodarowanie wodą w rolnictwie w obliczu ekstremalnych zjawisk pogodowych (Water management in agriculture in the face of extreme weather phenomena). Postępy Nauk Rol..

[59] Markonis Y., Kumar R., Hanel M., Rakovec O., Máca P., Kouchak A.A. (2021). The rise of compound warm-season droughts in Europe. Sci. Adv..

[60] Hanel M., Rakovec O., Markonis Y., Máca P., Samaniego L., Kyselý J., Kumar R. (2018). Revisiting the recent European droughts from a long-term perspective. Sci. Rep..

[61] De Bono A., Giuliani G., Kluster S., Peduzzi P. (2004). Impact of summer 2003 heat wave in Europe. Environ. Alert Bull. UNEP.

[62] Micale F., Voght J., Cammalleri C. (2015). Drought News August 2015. Eur. Drought Obs. (EDO).

[63] (2007). Water Scarcity & Droughts. In-Depth Assessment. Second Interim Report—June 2007.

[64] Havrlentová M., Kraic J., Gregusová V., Kovácsová B. (2021). Drought Stress in Cereals—A Review. Agriculture.

[65] Zhang J., Zhang S., Cheng M., Jiang H., Zhang X., Peng C., Lu X., Zhang M., Jin J. (2018). Effect of drought on agronomic traits of rice and wheat: A meta-analysis. Int. J. Environ. Res. Public Health.

[66] Fowler D.B., Limin A.E., Wang S.Y., Ward R.W. (1996). Relationship between low-temperature tolerance and vernalization response in wheat and rye. Can. J. Plant Sci..

[67] Yang L., Yang D., Yan X., Cui L., Wang Z., Yuan H. (2016). The role of gibberellins in improving the resistance of tebuconazole-coated maize seeds to chilling stress by microencapsulation. Sci. Rep..

[68] Ding Y., Shi Y., Yang S. (2019). Advances and challenges in uncovering cold tolerance regulatory mechanisms in plants. New Phytol..

[69] Seki M., Kamei A., Yamaguchi-Shinozaki K., Shinozaki K. (2003). Molecular responses to drought, salinity and frost: Common and different paths for plant protection. Curr. Opin. Biotechnol..

[70] Guy C.L. (1990). Cold acclimation and freezing stress tolerance: Role of protein metabolism. Annu. Rev. Plant Physiol. Plant Mol. Biol..

[71] Uemura M., Tominaga Y., Nakagawara C., Shigematsu S., Minami A., Kawamura Y. (2006). Responses of the plasma membrane to low temperatures. Physiol. Plant..

[72] Yamori W., Hikosaka K., Way D.A. (2014). Temperature response of photosynthesis in C3, C4, and CAM plants: Temperature acclimation and temperature adaptation. Photosynth. Res..

[73] Yadav S.K. (2010). Cold stress tolerance mechanisms in plants: A review. Agron. Sustain. Dev..

[74] Thomashow M.F. (1998). Role of cold-responsive genes in plant freezing tolerance. Plant Physiol..

[75] Nievola C.C., Carvalho C.P., Carvalho V., Rodrigues E. (2017). Rapid responses of plants to temperature changes. Temperature.

[76] Achard P., Gong F., Cheminant S., Alioua M., Hedden P., Genschika P. (2008). The cold-inducible CBF1 factor-dependent signaling pathway modulates the accumulation of the growth-repressing DELLA proteins via its effect on gibberellin metabolism. Plant Cell.

[77] Zhou M., Chen H., Wei D., Ma H., Lin J. (2017). Arabidopsis CBF3 and DELLAs positively regulate each other in response to low temperature. Sci. Rep..

[78] Qin D., Wu H., Peng H., Yao Y., Ni Z., Li Z., Zhou C., Sun Q. (2008). Heat stress-responsive transcriptome analysis in heat susceptible and tolerant wheat (Triticum aestivum L.) by using Wheat Genome Array. BMC Genom..

[79] Thussagunpanit J., Jutamanee K., Sonjaroon W., Kaveeta L., Chai-Arree W., Pankean P., Suksamrarn A. (2015). Effects of brassinosteroid and brassinosteroid mimic on photosynthetic efficiency and rice yield under heat stress. Photosynthetica.

[80] Źróbek-Sokolnik A., Ahmad P., Prasad M.N. (2012). Temperature stress and responses of plants. Environmental Adaptations and Stress Tolerance of Plants in the Era of Climate Change.

[81] Bita C.E., Gerats T. (2013). Plant tolerance to high temperature in a changing environment: Scientific fundamentals and production of heat stress-tolerant crops. Front. Plant Sci..

[82] Liu Y., Zhang M., Meng Z., Wang B., Chen M. (2020). Research progress on the roles of cytokinin in plant response to stress. Int. J. Mol. Sci..

[83] Ritonga F.N., Chen S. (2020). Physiological and molecular mechanism involved in cold stress tolerance in plants. Plants.

[84] Sakata T., Oda S., Tsunaga Y., Shomura H., Kawagishi-Kobayashi M., Aya K., Saeki K., Endo T., Nagano K., Kojima M. (2014). Reduction of gibberellin by low temperature disrupts pollen development in rice. Plant Physiol..

[85] Colebrook E.H., Thomas S.G., Phillips A.L., Hedden P. (2014). The role of gibberellin signalling in plant responses to abiotic stress. J. Exp. Biol..

[86] Rahman A. (2013). Auxin: A regulator of cold stress response. Physiol. Plant..

[87] Singh I., Kumar U., Singh S.K., Gupta C., Singh M., Kushwaha S.R. (2012). Physiological and biochemical effect of 24-epibrassinoslide on cold tolerance in maize seedlings. Physiol. Mol. Biol. Plants.

[88] Pociecha E., Dziurka M., Oklestkova J., Janeczko A. (2016). Brassinosteroids increase winter survival of winter rye (Secale cereale L.) by affecting photosynthetic capacity and carbohydrate metabolism during the cold acclimation process. Plant Growth Regul..

[89] Janeczko A., Pociecha E., Dziurka M., Jurczyk B., Libik-Konieczny M., Oklestkova J., Novak O., Pilarska M., Filek M., Rudolphi-Skórska E. (2019). Changes in content of steroid regulators during cold hardening of winter wheat—Steroid physiological/biochemical activity and impact on frost resistance. Plant Physiol. Biochem..

[90] Janeczko A., Oklešťková J., Pociecha E., Kościelniak J., Mirek M. (2011). Physiological effects and transport of 24-epibrassinolide in heat-stressed barley. Acta Physiol. Plant..

[91] Thussagunpanit J., Jutamanee K., Kaveeta L., Chai-arree W., Pankean P., Homvisasevongsa S., Suksamrarn A. (2015). Comparative effects of brassinosteroid and brassinosteroid mimic on improving photosynthesis, lipid peroxidation and rice seed set under heat stress. J. Plant Growth Regul..

[92] Fahad S., Hussain S., Saud S., Khan F., Hassan S., Nasim W., Arif M., Wang F., Huang J. (2016). Exogenously applied plant growth regulators affect heat-stressed rice pollens. J. Agron. Crop Sci..

[93] Machado S., Paulsen G.M. (2001). Combined effects of drought and high temperature on water relations of wheat and sorghum. Plant Soil.

[94] Pociecha E., Janeczko A., Dziurka M., Gruszka D. (2020). Disturbances in the biosynthesis or signalling of brassinosteroids that are caused by mutations in the HvDWARF, HvCPD and HvBRI1 genes increase the tolerance of barley to the deacclimation process. J. Plant Growth Regul..

[95] Rapacz M., Jurczyk B., Sasal M. (2017). Deacclimation may be crucial for winter survival of cereals under warming climate. Plant Sci..

[96] Janeczko A., Biesaga-Kościelniak J., Dziurka M., Filek M., Hura K., Jurczyk B., Kula M., Oklestkova J., Novak O., Rudolphi-Skórska E. (2018). Biochemical and physicochemical background of mammalian androgen activity in winter wheat exposed to low temperature. J. Plant Growth Regul..

[97] Wu C., Cui K., Wang W., Li Q., Fahad S., Hu Q., Huang J., Nie L., Peng S. (2016). Heat-induced phytohormone changes are associated with disrupted early reproductive development and reduced yield in rice. Sci. Rep..

[98] Janowiak F., Luck E., Dörffling K. (2003). Chilling tolerance of maize seedlings in the field during cold periods in spring is related to chilling-induced increase in abscisic acid level. J. Agron. Crop Sci..

[99] Wang Z.Y., Seto H., Fujioka S., Yochida S., Chory J. (2001). BRI1 is a critical component of a plasma-membrane receptor for plant steroids. Nature.

[100] Kinoshita T., Cano-Delgado A., Seto H., Hiranuma S., Fujioka S., Yoshida S., Chory J. (2005). Binding of brassinosteroids to the extracellular domain of plant receptor kinase BRI1. Nature.

[101] Kim B.K., Fujioka S., Takatsuto S., Tsujimoto M., Choe S. (2008). Castasterone is a likely end product of brassinosteroid biosynthetic pathway in rice. Biochem. Biophys. Res. Commun..

[102] Xu R., He Y., Wang Y., Zhao Y. (1994). Preliminary study of brassinosterone binding sites from mung bean epicotyls. Acta Phytophysiol. Sin..

[103] Filek M., Sieprawska A., Kościelniak J., Oklestkova J., Jurczyk B., Telk A., Biesaga-Kościelniak J., Janeczko A. (2019). The role of chloroplasts in the oxidative stress that is induced by zearalenone in wheat plants—The functions of 24-epibrassinolide and selenium in the protective mechanisms. Plant Physiol. Biochem..

[104] Efimova M.V., Kusnetsov V.V., Kravtsov A.K., Karnachuk R.A., Khripach V.A., Kuznetsov V.V. (2012). Regulation of the transcription of plastid genes in plants by brassinosteroids. Dokl. Biol. Sci..

[105] Rizza F., Pagani D., Stanca A.M., Cattivelli L. (2001). Use of chlorophyll fluorescence to evaluate the cold acclimation and freezing tolerance of winter and spring oats. Plant Breed..

[106] Rapacz M., Wolanin B., Hura K., Tyrka M. (2008). The effects of cold acclimation on photosynthetic apparatus and the expression of COR14b in four genotypes of barley (Hordeum vulgare) contrasting in their tolerance to freezing and high-light treatment in cold conditions. Ann. Bot..

[107] Pociecha E., Dziurka M., Waligórski P., Krępski T., Janeczko A. (2017). 24-epibrassinolide pre-treatment modifies cold-induced photosynthetic acclimation mechanisms and phytohormone response of perennial ryegrass in cultivar-dependent manner. J. Plant Growth Regul..

[108] Hassan M.A., Xiang C., Farooq M., Muhammad N., Yan Z., Hui X., Yuanyuan K., Bruno A.K., Lele Z., Jincai L. (2021). Cold Stress in Wheat: Plant Acclimation Responses and Management Strategies. Front. Plant Sci..

[109] Liu X., Feng Z.M., Zhou C.L., Ren Y.K., Mou C.L., Wu T., Yang C.Y., Liu S.J., Jiang L., Wan J.M. (2016). Brassinosteroid (BR) biosynthetic gene lhdd10 controls late heading and plant height in rice (Oryza sativa L.). Plant Cell Rep..

[110] Filek M., Rudolphi-Skórska E., Sieprawska A., Kvasnica M., Janeczko A. (2017). Regulation of the membrane structure by brassinosteroids and progesterone in winter wheat seedlings exposed to low temperature. Steroids.

[111] Ferguson J.N., McAusland L., Smith K.E., Price A.H., Wilson Z.A., Murchie E.H. (2020). Rapid temperature responses of photosystem II efficiency forecast genotypic variation in rice vegetative heat tolerance. Plant J..

[112] Balla K., Bencze S., Bónis P., Árendás T., Veisz O. (2014). Changes in the photosynthetic efficiency of winter wheat in response to abiotic stress. Open Life Sci..

[113] Sami F., Yusuf M., Faizan M., Faraz A., Hayat S. (2016). Role of sugars under abiotic stress. Plant Physiol. Biochem..

[114] Trischuk R.G., Schilling B.S., Low N.H., Gray G.R., Gusta L.V. (2014). Cold acclimation, de-acclimation and re-acclimation of spring canola, winter canola and winter wheat: The role of carbohydrates, cold-induced stress proteins and vernalization. Environ. Exp. Bot..

[115] Wu C., Trieu A., Radhakrishnan P., Kwok S.F., Harris S., Zhang K., Wang J., Wan J., Zhai H., Takatsuto S. (2008). Brassinosteroids regulate grain filling in rice. Plant Cell.

[116] Janeczko A., Gruszka D., Pociecha E., Dziurka M., Filek M., Jurczyk B., Kalaji H.M., Kocurek M., Waligórski P. (2016). Physiological and biochemical characterisation of watered and drought-stressed barley mutants in the HvDWARF gene encoding C6-oxidase involved in brassinosteroid biosynthesis. Plant Physiol. Biochem..

[117] Mathur S., Agrawal D., Jajoo A. (2014). Photosynthesis: Response to high temperature stress. J. Photochem. Photobiol. B Biol..

[118] Ben-Asher J., Garcia Y., Garcia A., Hoogenboom G. (2008). Effect of high temperature on photosynthesis and transpiration of sweet corn (Zea mays L. var. rugosa). Photosynthetica.

[119] Djanaguiraman M., Boyle D.L., Welti R., Jagadish S.V.K., Prasad P.V.V. (2018). Decreased photosynthetic rate under high temperature in wheat is due to lipid desaturation, oxidation, acylation, and damage of organelles. BMC Plant Biol..

[120] Zhao G., Xu H., Zhang P., Su X., Zhao H. (2017). Effects of 2,4-epibrassinolide on photosynthesis and Rubisco activase gene expression in Triticum aestivum L. seedlings under a combination of drought and heat stress. Plant Growth Regul..

[121] Prasad M.N.V., Rengel Z. (2006). Plant acclimation and adaptation to natural and anthropogenic stress. Ann. N. Y. Acad. Sci..

[122] Horváth I., Glatz A., Nakamoto H., Mishkind M.L., Munnik T., Saidi Y., Goloubinoff P., Harwood J.L., Vigh L. (2012). Heat shock response in photosynthetic organisms: Membrane and lipid connections. Prog. Lipid Res..

[123] Larkindale J., Huang B. (2004). Changes of lipid composition and saturation level in leaves and roots for heat-stressed and heat-acclimated creeping bentgrass (Agrostis stolonifera). Environ. Exp. Bot..

[124] Narayanan S., Tamura P.J., Roth M.R., Prasad P.V.V., Welti R. (2016). Wheat leaf lipids during heat stress: I. High day and night temperatures result in major lipid alterations. Plant Cell Environ..

[125] Gzyl-Malcher B., Zembala M., Filek M. (2010). Effect of tocopherol on surface properties of plastid lipids originating from wheat calli cultivated in cadmium presence. Chem. Phys. Lipids.

[126] Rochester C.P., Kjellbom P., Andersson B., Larsson C. (1987). Lipid composition of plasma membranes isolated from light-grown barley (Hordeum vulgare) leaves: Identification of cerebroside as a major component. Arch. Biochem. Biophys..

[127] Boudière L., Michaud M., Petroutsos D., Rébeillé F., Falconet D., Bastien O., Roy S., Finazzi G., Rolland N., Jouhet J. (2014). Glycerolipids in photosynthesis: Composition, synthesis and trafficking. Biochim. Biophys. Acta—Bioenerg..

[128] Apdila E.T., Awai K. (2018). Configuration of the sugar head of glycolipids in thylakoid membranes. Genes Genet Syst..

[129] Dufourc E.J. (2008). Sterols and membrane dynamics. J. Chem. Biol..

[130] Fujii S., Wada H., Kobayashi K. (2019). Role of Galactolipids in Plastid Differentiation Before and After Light Exposure. Plants.

[131] Oren I., Fleishman S.J., Kessel A., Ben-Tal N. (2004). Free diffusion of steroid hormones across biomembranes: A simplex search with implicit solvent model calculations. Biophys. J..

[132] Vijayan R., Biggin P.C. (2008). A steroid in a lipid bilayer: Localization, orientation, and energetics. Biophys. J..

[133] Li B., Zhang C., Cao B., Qin G., Wang W., Tian S. (2012). Brassinolide enhances cold stress tolerance of fruit by regulating plasma membrane proteins and lipids. Amino Acids.

[134] Rudolphi-Szydło E., Dyba B., Janeczko A., Latowski D., Sadura I., Filek M. (2021). Brassinosteroid-lipid membrane interaction under low and high temperature stress in model systems. BMC.

[135] Sadura I. (2014). Izolacja i oczyszczanie barwników cyklu diadinoksantynowego i ich wpływ na dynamikę molekularną błon modelowych (Isolation and purification of diadinoxanthin cycle pigments and their influence on molecular dynamic of artificial membranes). Master’s Thesis.

[136] Park C.J., Seo Y.S. (2015). Heat shock proteins: A review of the molecular chaperones for plant immunity. Plant Pathol. J..

[137] Ul Haq S., Khan A., Ali M., Khattak A.M., Gai W.X., Zhang H.X., Wei A.M., Gong Z.H. (2019). Heat shock proteins: Dynamic biomolecules to counter plant biotic and abiotic stresses. Int. J. Mol. Sci..

[138] Kapilan R., Vaziri M., Zwiazek J.J. (2018). Regulation of aquaporins in plants under stress. Biol. Res..

[139] Ambroise V., Legay S., Guerriero G., Hausman J.F., Cuypers A., Sergeant K. (2020). The roots of plant frost hardiness and tolerance. Plant Cell Physiol..

[140] Beck E.H., Fettig S., Knake C., Hartig K., Bhattarai T. (2007). Specific and unspecific responses of plants to cold and drought stress. J. Biosci..

[141] Afzal Z., Howton T.C., Sun Y., Mukhtar M.S. (2016). The roles of aquaporins in plant stress responses. J. Dev. Biol..

[142] Sze H., Li X., Palmgren M.G. (1999). Energization of plant cell membranes by H + -pumping ATPases: Regulation and biosynthesis. Plant Cell.

